# Brain-wide 3D neuron detection and mapping with deep learning

**DOI:** 10.1117/1.NPh.12.2.025012

**Published:** 2025-05-20

**Authors:** Yuanyang Liu, Ziyan Gao, Zhehao Xu, Chaoyue Yang, Pei Sun, Longhui Li, Hongbo Jia, Xiaowei Chen, Xiang Liao, Junxia Pan, Meng Wang

**Affiliations:** a Chongqing University, School of Medicine, Center for Neurointelligence, Chongqing, China; b Third Military Medical University, Brain Research Center, State Key Laboratory of Trauma and Chemical Poisoning, Chongqing, China; c Chinese Academy of Sciences, Suzhou Institute of Biomedical Engineering and Technology, Brain Research Instrument Innovation Center, Suzhou, China; d Chongqing Institute for Brain and Intelligence, Guangyang Bay Laboratory, Chongqing, China; e Chongqing Medical University, Institute for Brain Science and Disease, Chongqing, China

**Keywords:** whole-brain imaging, neuron mapping, 3D soma detection, deep learning, video swin transformer

## Abstract

**Significance:**

Mapping the spatial distribution of specific neurons across the entire brain is essential for understanding the neural circuits associated with various brain functions, which in turn requires automated and reliable neuron detection and mapping techniques.

**Aim:**

To accurately identify somatic regions from 3D imaging data and generate reliable soma locations for mapping to diverse brain regions, we introduce NeuronMapper, a brain-wide 3D neuron detection and mapping approach that leverages the power of deep learning.

**Approach:**

NeuronMapper is implemented as a four-stage framework encompassing preprocessing, classification, detection, and mapping. Initially, whole-brain imaging data is divided into 3D sub-blocks during the preprocessing phase. A lightweight classification network then identifies the sub-blocks containing somata. Following this, a Video Swin Transformer–based segmentation network delineates the soma regions within the identified sub-blocks. Last, the locations of the somata are extracted and registered with the Allen Brain Atlas for comprehensive whole-brain neuron mapping.

**Results:**

Through the accurate detection and localization of somata, we achieved the mapping of somata at the one million level within the mouse brain. Comparative analyses with other soma detection techniques demonstrated that our method exhibits remarkably superior performance for whole-brain 3D soma detection.

**Conclusions:**

Our approach has demonstrated its effectiveness in detecting and mapping somata within whole-brain imaging data. This method can serve as a computational tool to facilitate a deeper understanding of the brain’s complex networks and functions.

## 1 Introduction

To comprehend the neural circuits that underlie computations in the brain, it is essential to map individual, labeled neurons across the brain structure, which is one of the major challenges in contemporary neuroscience.^1^^–^^3^ As the exact location and spatial arrangement of neurons are intrinsically linked to brain functions, it is crucial to achieve accurate neuron localization, particularly at a whole-brain scale.^4^ The advancements in labeling and optical imaging methodologies have enabled the monitoring of the brain which contains millions of neurons.^5^^–^^7^ However, even if only 1% of these neurons are labeled, the manual annotation becomes extremely laborious and prone to error.^8^ Computational approaches provide a promising avenue by automating the detection of neurons, thus accelerating the neuron mapping process.^9^ Nevertheless, the complexities inherent in large-scale optical imaging datasets—such as brightness fluctuations within 3D images, the diverse morphology of neurons, and the frequent overlap of somata with indistinct boundaries—pose significant obstacles to automated processing. These complexities highlight the urgent necessity to develop robust and accurate tools for automatic soma detection, which is crucial for advancing whole-brain neuronal mapping efforts and will play a critical role in dissecting the brain’s mechanisms.

In previous studies, a series of traditional methods and tools have been developed to detect somata in biomedical images. Intensity threshold is used when there are notable grayscale differences between somata and the background.^10^ Static threshold is applied in cell segmentation for extracting soma regions.^11^ The OTSU threshold determines the grayscale histogram of the image based on an appropriate setting to extract the foreground of the image.^12^ OTSU is generally efficient for intensity-based thresholding, but it tends to perform poorly in the presence of data imbalance, outliers, and artifacts. However, these methods are mainly confined to 2D images and yield limited results. Although some methods excel at identifying labeled cells in serial 2D sections, 2D analysis can introduce bias due to potential under- or overestimation of cell numbers based on sampling within the third dimension. The 3D analysis methods inherently address this limitation by accounting for the full spatial context. For example, Quan et al.^13^ proposed NeuroGPS to extract morphological features for initial detection and combine L1 to minimize the foreground of modeled images via a spherical model (performance: true positive rate of 88%; false positive rate of 8%). Hu et al.^14^ proposed an automatic soma segmentation method, based on the Rayburst sampling algorithm, which is suitable for datasets featuring touching or overlapping somata (performance: precision of 0.965; recall of 0.975). In addition, He et al.^15^ initially achieved the soma detection results through distance transformation and then screened the results through a logistic regression model, which performed well in fruit-fly brain datasets (performance: sensitivity of 0.918; accuracy of 0.844; F1-score of 0.872). Despite these progressions, their detection methods remain basic, dataset-specific, and lack efficiency for those with dense somata.

Recent advancements in deep learning have shed light on new avenues for enhancing biological image processing. Convolutional neural networks (CNNs) have been extensively utilized in biomedical image processing owing to their capability to automatically learn multilevel features from raw data. The application of appropriate data augmentation techniques, such as random translations, rotations, and scaling, imparts CNNs with invariance to these transformations. This invariance has been key to achieving robust performance across various applications.^16^ For neuronal soma segmentation and detection tasks, some promising results have been achieved in previous studies. Among them, U-Net, a prominent CNN architecture, has performed exceptionally well in segmenting complex neuronal structures from electron microscopy images (performance: intersection over union of 0.9163; accuracy of 0.9779).^17^ Stringer et al.^18^ proposed Cellpose, a deep learning–based method that segments cells from a wide range of image types without requiring model retraining or parameter adjustments (performance: average precision of 0.91). Li et al.^19^ utilized CNNs to classify the presence of somata, advancing the efforts in whole-brain data categorization. Tyson et al.^9^ developed a pipeline that involves the application of a deep neural network to distinguish true cells and to accelerate the analysis (performance: Pearson correlation coefficient of 0.999, algorithm versus expert). Dong et al.^20^ developed a deep supervised network and a novel training strategy for soma segmentation at single-neuron resolution (performance: Jaccard of 0.6811; Dice of 0.7910; precision of 0.7870; recall of 0.8329). Hu et al.^21^ achieved neuronal soma segmentation using 3D multitask learning with U-shaped fully convolutional neural networks (performance: precision of 0.94; recall of 0.90; F1-score of 0.92; Dice coefficient of 0.8436). Wei et al.^22^ developed two-stage deep neural networks for soma detection in large, high-resolution whole mouse brain images (performance: precision of 0.9812; recall of 0.891; F1-score of 0.934). However, these CNN-based networks, which are designed for sparsely distributed somata, may yield less than ideal results when dealing with densely distributed somata due to overlapping structures. Therefore, there remains a crucial need for methods specifically tailored to dense soma images. A significant challenge in deep learning–based approaches is the effective encoding of long-range dependencies, which is hindered by the restricted receptive fields of convolution kernels.^23^ This limitation undermines dense prediction tasks and potentially leads to a loss of essential global information.^24^^,^^25^

To deal with the challenges of accurately detecting somata and effectively analyzing 3D whole-brain datasets for comprehensive neuronal mapping, we present a brain-wide 3D neuron detection and mapping method (NeuronMapper). Our approach integrates preprocessing, classification, detection, and mapping modules, which are designed to streamline and optimize the entire processing workflow. In our pipeline, the classification module has a crucial role in reducing data redundancy by selectively filtering out irrelevant data blocks from the whole-brain dataset. This processing step ensures that only relevant information is passed on to the subsequent stages. Following this, the detection module uses a Video Swin Transformer to deal with long-range dependency issues and segment the somatic regions. By analyzing the connected domains defined by these segments and determining the center points as the locations of the somata, we establish a robust framework for neuron detection and mapping. The coordinates derived from individual sub-blocks are then aggregated, enabling the construction of a comprehensive, whole-brain neuronal map. This foundational mapping supports quantitative analysis in specific functional circuit studies and provides an integrated view of upstream and downstream neural circuitry, thereby advancing our understanding of brain networks.^26^^,^^27^

## 2 Materials and Methods

### 2.1 Experimental Dataset

#### 2.1.1 Animals and surgery

C57BL/6J male mice (aged 8 to 12 weeks) were obtained from the Laboratory Animal Center, Third Military Medical University. These animals were housed in a temperature- and humidity-controlled room on a cycle of 12-h light/dark (lights off at 19:00). All experimental procedures were carried out in accordance with the institutional animal welfare guidelines with the approval of the Third Military Medical University Animal Care and Use Committee. In the experiments, glass electrodes with a tip diameter of 20 to 30  μm filled with AAV2/2Retro Plus-hSyn-nuclear-EGFP were inserted, and ∼200  μL was slowly injected into the auditory cortex. Thirty days after the injection, the animals were anesthetized, perfused, and fixated.

#### 2.1.2 Fluorescence micro-optical sectioning tomography data acquisition and preprocessing

The preparation of brain specimens for fluorescence micro-optical sectioning tomography (fMOST) imaging was carried out following the Resin/HM20 Embedding Protocol.^28^ Basically, the samples were rinsed with PBS solution three times and dehydrated with ethanol solution at 4°C in the dark. Subsequently, the samples were penetrated with resin and quenched with acetic acid. The resin polymerization took place at 50°C for 8 h. For mouse whole-brain imaging, the embedded brain samples were imaged by the fMOST system at a resolution of 0.35  μm×0.35  μm×2  μm. Image preprocessing procedures encompassed image stitching, brightness adjustment, and noise filtering to improve the signal-to-noise ratio of the soma data from the GFP channel. We transformed the data into cuboid format via TDat2017 software,^29^ facilitating the ease of manipulation and analysis in subsequent computational workflows.

### 2.2 Neuron Detection and Mapping

#### 2.2.1 Overall process of analyzing whole-brain imaging data

We present a deep learning–based approach (NeuronMapper) for the detection and mapping of somata within whole-brain images, which is implemented as a four-stage framework, including preprocessing, classification, detection, and mapping. Initially, the three-dimensional brain images undergo a strategic partitioning process, dividing it into a series of smaller, analyzable sub-blocks. Here, the maximum intensity projection (MIP) was utilized to transform 3D image data into a single 2D image by projecting the brightest pixel (voxel) from each layer of the stack onto the final image. This process highlights the most intense signals within the original 3D image data. For each sub-block, an MIP image is calculated, which is a preparatory step that facilitates efficient parallel processing and optimizes the computational workflow for subsequent analysis. The classification processing is inspired by the previous study,^22^ and it plays a crucial role by sifting through the sub-blocks to exclude those without detectable somata, thereby significantly reducing the dataset size for the segmentation stage. This selective filtering ensures that only sub-blocks containing somata are forwarded for further processing. The segmentation network then utilizes Video Swin Transformer–based algorithms to outline the boundaries of somata, providing a precise morphological delineation. Following segmentation, a key step involves the application of a connected domain analysis to determine the centroid coordinates for each detected soma. This process enables us to accurately identify the location of each soma within the complex topology of the brain, contributing to a comprehensive understanding of neuron distribution. Through the proposed method, robust and precise detection of neuronal somata can be achieved throughout the entire brain, resulting in detailed soma locations. Finally, the combination of the coordinates and Allen Brain Atlas produces a comprehensive mapping of the somata in various brain regions. This facilitates a deep insight into the spatial arrangement and connectivity of neurons within the brain. Comprehensive elucidations of the algorithms used in the classification, segmentation, and mapping stages are provided in Secs. 2.2.2–2.2.6.

#### 2.2.2 Neuronal soma identification using the classification network

The preprocessing stage divided whole-brain image data into sub-blocks and processing them directly through detection would incur unnecessary computational expenses. To strategically avoid this inefficiency, we design a classification network that serves as a gatekeeper, selecting sub-blocks containing soma information while eliminating those lacking such information. Our approach initiates with the augmentation of the channel dimension through a convolutional layer, laying the foundation for feature extraction. Four successive stages, each composed of a convolutional layer, strided convolution, and a shortcut link, are sequentially employed to reduce the parameters while simultaneously capturing the essential characteristics of the images. Contrary to the conventional methods that advocate the use of max pooling layers, our method adopts convolutional layers with a stride of two. This application not only reduces the feature dimensions but also learns features efficiently and preserves spatial information. An average pooling layer is then placed to further compress the data, reducing the dimensionality of the feature map to a more manageable size. Except for the output layer, every convolutional layer is paired with a batch normalization layer and activated through the rectified linear unit (ReLU), a combination that facilitates the model’s rapid and stable convergence. The multidimensional features learned by the convolutional layer are converted into one-dimensional features using the fully connected layer. Finally, the Softmax function is used to calculate the likelihood of each image belonging to a specific class, resulting in the generation of definite classification results (Fig. S1 in the Supplementary Material).

#### 2.2.3 3D soma segmentation network

Following the classification stage, we design a segmentation network for separating the somatic regions from the remaining constituents of the image. The network architecture, which is shown in Fig. S2(a) in the Supplementary Material, is devised based on the Video Swin Transformer. This design starts with a series of convolutional layers, stacked to extract a detailed range of local features, followed by the strategic use of strided convolution to further refine these features. The resulting features are adjusted to meet the specifications of the transformer layer. Within the transformer layers, these features engage in a dynamic interaction, absorbing comprehensive global context and establishing intricate relationships across the entire image block. This enables the module to perceive the somatic regions not just as isolated entities but as integral parts of the broader spatial environment. Once exiting the transformer layers, the output undergoes a transformative upscaling process, returning to its original resolution through an up-sampling process. Concurrently, through the use of skip connections, the output is seamlessly merged with the corresponding encoder layers’ outputs, orchestrating a harmonious flow of information. This step-by-step restoration of resolution ensures that the fine balance between local detail and global perspective is maintained throughout the segmentation process. Each convolutional layer is enhanced with batch normalization to regularize the inputs and supported by the ReLU activation function, which introduces nonlinearity and facilitates feature learning. This design enables the network’s ability to seamlessly integrate local and global information, significantly enhancing the precision of the segmentation outcome. The distinct components in the segmentation module are described in the following.

For the feature extraction, the input is a 3D image sub-block, denoted as $F\in R^{C\times H\times W\times D}$, where it has a spatial resolution of H (height) × W (width), D is the depth dimension, and C is the number of channels. The initial convolutional layer maps the number of channels of the original data to the K-dimension, denoted as $F\in R^{K\times H\times W\times D}$. After the first stage of the encoder layer, the dimension remains unchanged, and then, the down-sampling effect is achieved through convolution with a step size of 2, and the size of the three dimensions of the feature is halved, denoted as $F\in R^{K\times \frac{H}{2}\times \frac{W}{2}\times \frac{D}{2}}$. The second and third stages are both constituted by stacking two encoders and one convolution with a step size of 2, and the size of the feature is reduced while the input data is encoded layer-by-layer into low-resolution, high-level features, which are represented as $F\in R^{K\times \frac{H}{8}\times \frac{W}{8}\times \frac{D}{8}}$. The data are down-sampled three times, and the size of the feature is one-eighth of the dimension of the original input. After the fourth stage of the four-layer convolutional encoders, the feature details are further extracted, and the dimensions do not change. Each data input is composed of a K-dimensional feature vector, and the rich local spatial context features are effectively embedded into the vector F after being processed by the feature extraction layers, which is represented as $F^{\prime }\in R^{K\times H^{\prime }\times W^{\prime }\times D^{\prime }}$.

The transformer layer is designed to capture both localized and global contextual information, enabling enhanced feature representation for 3D soma segmentation. The transformer layer is composed of L Video Swin Transformers, through two consecutive multihead self-attention layers and the self-attention. The number of Video Swin Transformer layers impacts both the model’s feature-capturing capabilities and its computational complexity. A greater number of layers improve the model’s ability to capture global features and enhance its expressiveness, which is beneficial for complex tasks such as neuron segmentation that involve intricate spatial structures. However, deeper networks can lead to overfitting and require more computational resources. Conversely, fewer layers emphasize local feature extraction, reducing computational demands and increasing efficiency, which is advantageous for real-time applications or scenarios with limited resources. In our model, we set L to 1, achieving a balance between performance and efficiency. This design efficiently captures the essential features for 3D soma segmentation while minimizing computational overhead and reducing the risk of overfitting. The comparative results for different settings of L are illustrated in Fig. S2(b) in the Supplementary Material. In the structure of Video Swin Transformers, the first layer uses the conventional window partitioning strategy to obtain nonoverlapping 3D windows. The second layer shifts the window according to a certain step size and then completes the self-attention processing. Using $F^{\prime }$ as the input of the transformer layer, the process applies layer normalization before setting the window size to ΔH×ΔW×ΔD, dividing the number of windows to $\frac{H}{\Delta H}\times \frac{W}{\Delta W}\times \frac{D}{\Delta D}$, and representing the window as $f^{\prime }\in R^{K^{\prime }\times \Delta H\times \Delta W\times \Delta D}$. Then, the window-based multihead self-attention/shifted-window-based multihead self-attention (W-MSA/SW-MSA) operates within each 3D window, where W-MSA stands for window multi-head self-attention module, and SW-MSA represents shifted window-based multihead self-attention module. The feedforward processing is then applied, and the residual connection is applied after each processing. For the second layer, the output obtained by the previous layer is taken as the input of the SW-MSA layer, and windows are moved along the axes of height, width, and depth $\left(\frac{\Delta H}{2},\frac{\Delta W}{2},\frac{\Delta D}{2}\right)$, and the other parts are consistent with the first layer.

The preceding encoder layer learns and extracts both local and global features. To obtain the ultimate spatial prediction results of the 3D data block, the up-sampling and restoration of the high-resolution images are completed by 3D convolution and the skip connection in the decoder. First, the channel dimension is reduced to be consistent with the input through two convolutional layers, and the feature map is reduced to a K-dimensional feature, which is represented as $F^{\prime }(K,\frac{H}{8},\frac{W}{8},\frac{D}{8})$. In the first stage, the output obtained in the third stage of feature extraction and the output of the restoration are concatenated by skip connection. In the second stage and the third stage, the feature is gradually restored to a high-resolution image through the decoder layer, and then the segmentation is completed.

#### 2.2.4 Soma detection using connected domain analysis

Following the segmentation stage in which the soma regions are outlined, the segmented regions themselves are utilized as the connectivity domain. Within this domain, the centroid of each somatic region is calculated, representing the most likely location of the soma. An analysis is conducted to compare the predicted soma centroids with those manually labeled, using a predefined threshold as the acceptance criterion for the soma centroid matches. The threshold was determined by calculating the average radius of the somata, which resulted in a threshold value of 10 pixels. If the Euclidean distance, i.e., the straight-line distance in the three-dimensional space, between the predicted and labeled points is below this established threshold, the detection of the soma is confirmed as a successful identification. This algorithm ensures that the performance of the detection algorithm is examined and validated with ground-truth data, thereby confirming its effectiveness and precision.

#### 2.2.5 Registration and quantitative analysis of the detected soma

The whole-brain imaging data were registered to the Allen Common Coordinate Framework.^30^ The registration process starts with the correction of spatial distortions in the imaged brains through a rigid registration approach. This is followed by a grayscale-guided, three-dimensional affine registration that aligns the imaging data with the Allen Brain Atlas. To further enhance the registration accuracy, particularly in more complex and localized regions, a dense landmark-based two-dimensional registration technique is adopted for focusing on specific areas to ensure an optimal fit. The Elastix software is utilized to carry out all registration steps, providing a framework for the alignment process. Once the registration is completed, the coordinates of the detected somata are transformed into the Allen Brain Atlas coordinate system, guided by the transformation parameters obtained during the registration stage. To ensure the reliability of the registration results, two experienced analysts independently evaluated the alignment across various mouse brain regions. They then reviewed any discordant results and discussed them to reach a final consensus (a back-to-back process), thereby ensuring the highest level of accuracy. Following this validation, neuron coordinates were systematically assigned to their respective brain regions, enabling an automated counting of neurons within each subregion. To provide a comparison of neuronal density and connection strength across different brain regions, the neuron counts were normalized by calculating the proportion of neurons in each subregion relative to the total number of neurons in the whole brain. This standardization facilitated a comprehensive analysis of neural connectivity patterns and densities, providing deep insights into the brain’s complex neural architecture and functional organization.

#### 2.2.6 Neural network model training and testing

Our approach was assessed on a 3D whole-brain imaging dataset; the model training included the classification network and the segmentation network. In the training stage, the model was constructed using Python 3.6 within the PyTorch 1.10.2 framework and was trained on an NVIDIA RTX A5000 GPU. For the image classification, soma segmentation, and detection tasks, two experienced annotators independently labeled each image and soma within the dataset. They subsequently compared their annotations to resolve any discrepancies, reaching a final consensus that established a reliable ground truth (GT). This GT was then used for training and testing the methods, providing a consistent and accurate benchmark for performance evaluation. For training the classification network, the dataset consisted of 180 images derived from four different mice. Positive samples were manually selected that covered diverse cases, including various instances of soma sub-blocks such as different densities, brightness levels, and distribution locations. Negative samples were randomly selected from the background images. The dataset was randomly divided into three sub-datasets: the training dataset (120 images), the validation dataset (30 images), and the testing dataset (30 images). We randomly repeated the data assignment to the datasets 10 times to validate the stability of our classification network. We trained the four methods (our classification network, ResNet18, VGG16, and DenseNet121) for 100 epochs. The classification network was trained using the stochastic gradient descent optimizer with an initial learning rate of 0.0001 and a minimum batch size of 8. To enhance the model’s generalization and prevent overfitting, an early stopping strategy was employed. Specifically, after each epoch, the area under the curve (AUC) on the validation dataset was calculated and monitored. Training was halted early if there was no significant improvement in the validation AUC for 10 consecutive epochs [Fig. S3(a) in the Supplementary Material].

During the model training stage of segmentation, the dataset was divided into three sub-datasets from two different mice: two training datasets (22 blocks per dataset), two validation datasets (6 blocks per dataset), and two testing datasets (10 blocks per dataset). Two-round cross-validation was conducted by selecting sub-datasets from the mouse imaging data, ensuring separate training, validation, and testing datasets for each round. In this study, we evaluated seven deep learning–based methods: our segmentation network, SomaSegmenter, Cellpose, SegNet, 3D U-Net, ResUNet, and TransBTS. Each method was trained from scratch without pretrained weights, using a dynamic learning rate and a training duration of 200 epochs. The Adam optimizer with an initial learning rate of 0.0001 was used to train the segmentation network. The learning rate decay strategy was adopted, and the learning rate decayed by 10% every 10 epochs. The best model was obtained using the early stopping strategy, where training was stopped if the Dice coefficient did not improve over 30 consecutive epochs [Fig. S3(b) in the Supplementary Material]. Each epoch contained 80 iterations and the minimum batch size was set to 1 in the segmentation network training stage. Dice loss was used in the training network task.

During the training stage, a comprehensive data augmentation pipeline was applied to the input images to enhance sample diversity and improve the robustness of the segmentation network. This pipeline comprised a random crop operation, a left-right random flip, and an up-down random flip. The random crop operation extracted fixed-size patches with a specified crop size of 128 pixels. Both the left-right and up-down flips were applied with a probability of 50% each. To maintain the spatial correspondence between images and their respective segmentation labels, these transformations were simultaneously applied to both.

In the testing phase, preprocessing was applied to the input images to optimize the segmentation network’s performance. The process began with resampling to standardize image resolution, ensuring all inputs have a uniform scale despite potential variations in their original resolutions. Images were resized by applying down-sampling factors to the Z-axis (depth) and X, Y axes (width and height), aligning the dimensions with the model’s input requirements. Mathematically, this involves scaling each dimension by a factor that reduces computational load while preserving key features. Next, padding was added to the images to ensure compatibility with the convolutional layers of the network. By adding zeros around the edges, padding maintains the alignment of convolutional operations with the image boundaries, thereby preventing edge information loss. The padding size is calculated to make the image dimensions multiples of the stride length, allowing for consistent processing across the entire image. To manage large images efficiently and avoid memory overflow, patch extraction was employed. The image was divided into smaller, overlapping patches based on specified patch size and stride parameters. The sliding window technique used for patch extraction ensures that critical features at the boundaries are captured through the overlap, whereas the number of patches is determined by how many strides fit within the padded image dimensions. This approach enables efficient handling of large images without losing important boundary information.

### 2.3 Performance Evaluation

To quantify classification performance, the AUC, accuracy, sensitivity, and specificity were used as the metrics to evaluate the methods.^31^

$$\text{Accuracy}=\frac{\mathrm{TP}+\mathrm{TN}}{\mathrm{TP}+\mathrm{TN}+\mathrm{FP}+\mathrm{FN}},$$ (1)

$$\text{Sensitivity}=\frac{\mathrm{TP}}{\mathrm{TP}+\mathrm{FN}},$$ (2)

$$\text{Specificity}=\frac{\mathrm{TN}}{\mathrm{TN}+\mathrm{FP}},$$ (3)

where true positive (TP) regions refer to the number of cases where positives are correctly predicted, true negative (TN) regions refer to the number of cases where negatives are correctly predicted, false positive (FP) regions indicate the number of cases incorrectly predicted as positive, and false negative (FN) regions denote the number of cases incorrectly predicted as negative.

To quantitatively analyze segmentation accuracy, Dice similarity coefficient (DSC) and average Euclidean distance were used to evaluate the methods.^32^ The metrics are defined as follows:

$$\mathrm{DCS}=\frac{2|S_{g}\cap S_{t}|}{\left|S_{g}|+|S_{t}\right|},$$ (4)

where $S_{t}$ and $S_{g}$ represent the regions inside the automatic segmentation and the manually marked segmentation (ground truth), respectively.

$$\text{Average Euclidean Distance}=\frac{1}{N}\overset{N}{\underset{i=1}{\sum }}\;\min_{v_{2}\in pre,{\left\|v_{1,i}-v_{2,i}\right\|}_{2}\leq diff}{\left\|v_{1,i}-v_{2,i}\right\|}_{2},$$ (5)

$${\left\|v_{1,i}-v_{2,i}\right\|}_{2}=\sqrt{\overset{d}{\underset{i=1}{\sum }}(v_{1,i}-v_{2,i}{)}^{2}},$$ (6)

where N represents the number of matched points, pre represents the prediction results, diff is the specified threshold, $v_{1,i}$ represents the i’th point in the ground truth dataset, and $v_{2,i}$ represents the corresponding matched point in the prediction dataset. During the calculation, the distance between $v_{1}$ and each point in pre is computed, and the point that satisfies the threshold condition is identified as a candidate match. If multiple points satisfy the threshold, the point with the minimum distance is selected as the unique match.

Three metrics (precision, recall, and F1-score) were used for the evaluation of the detection performance:^33^

$$\text{Precision}=\frac{N_{\mathrm{TP}}}{N_{\mathrm{GT}}},$$ (7)

$$\text{Recall}=\frac{N_{\mathrm{TP}}}{N_{\text{detected}}},$$ (8)

$$F1=\frac{2\times \text{Recall}\times \text{Precision}}{\text{Recall}+\text{Precision}},$$ (9)

where $N_{\mathrm{GT}}$ is the number of ground truth somata, $N_{\mathrm{TP}}$ is the number of true-positive somata, and $N_{\text{detected}}$ is the number of detected somata.

### 2.4 Statistical Analysis

In the results, the summarized data are presented as the mean ± *SD*. To compare data between two groups, we used a two-sided Wilcoxon signed-rank test to determine statistical significance, with a threshold of p<0.05 for the statistical difference. All data processing in this study was performed using scripts written in Python.

For further statistical information regarding the experiments, please refer to the figure legends.

## 3 Results

### 3.1 Brain-Wide Imaging of Individual Neuronal Somata

**Fig. 1:**
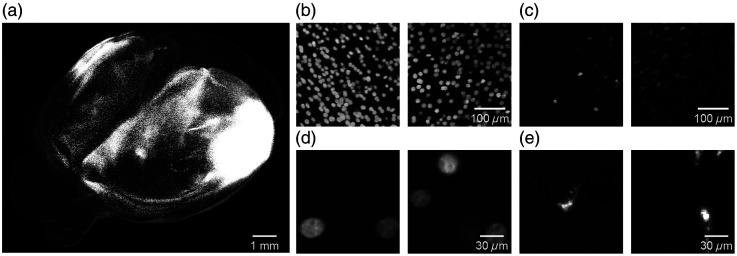
Examples of somata and background noises in the whole-brain imaging data. (a) An example of whole-brain imaging data that contains a large number of somata. (b) Sub-blocks having densely distributed somata. (c) Sub-blocks having somata with diverse fluorescence intensity. (d) Representative somata within the sub-blocks. (e) Representative background noises within the sub-blocks.

The neocortex of mammal functions as a central processor, handling complex neuronal signals within localized microcircuits and promoting the synthesis of information across diverse brain layers and regions. To elucidate the complexity and to demonstrate the capabilities of neuronal mapping, we utilized whole-brain images from mice that had undergone retrograde viral labeling. The experimental data were acquired from four male C57BL/6 mice, aged 11 to 12 weeks, which were labeled using AAV2/2Retro Plus-hSyn-nuclear-EGFP and imaged using the fMOST imaging system. The detailed experimental procedure is provided in Sec. 2. Figure 1 shows a visualization of the soma distribution patterns obtained in a representative mouse [Fig. 1(a)], highlighting the areas with densely distributed somata [Fig. 1(b)] and the variability in fluorescence intensity among somata [Fig. 1(c)]. Representative somata and instances of background noise are depicted in Figs. 1(d) and 1(e), respectively. Certain sub-blocks without somata may contain noises that closely resemble somata, potentially leading to misclassification. It should be noted that the 3D imaging of the whole mouse brain with single-neuron resolution produces several terabytes of image data. To address this issue and to standardize the imaging data for further processing, cubic interpolation was employed to resize the raw 0.35×0.35×2  μm image blocks. Subsequently, these blocks were subdivided into smaller sub-blocks (512×512×512  voxels) with a standardized spatial resolution of 0.35×0.35×0.35  μm/voxel, ensuring uniform voxel dimensions across the dataset. Comprehensive details about the imaging dataset can be found in Table S1 in the Supplementary Material.

### 3.2 Pipeline of the Proposed Neuron Detection and Mapping Approach

**Fig. 2:**
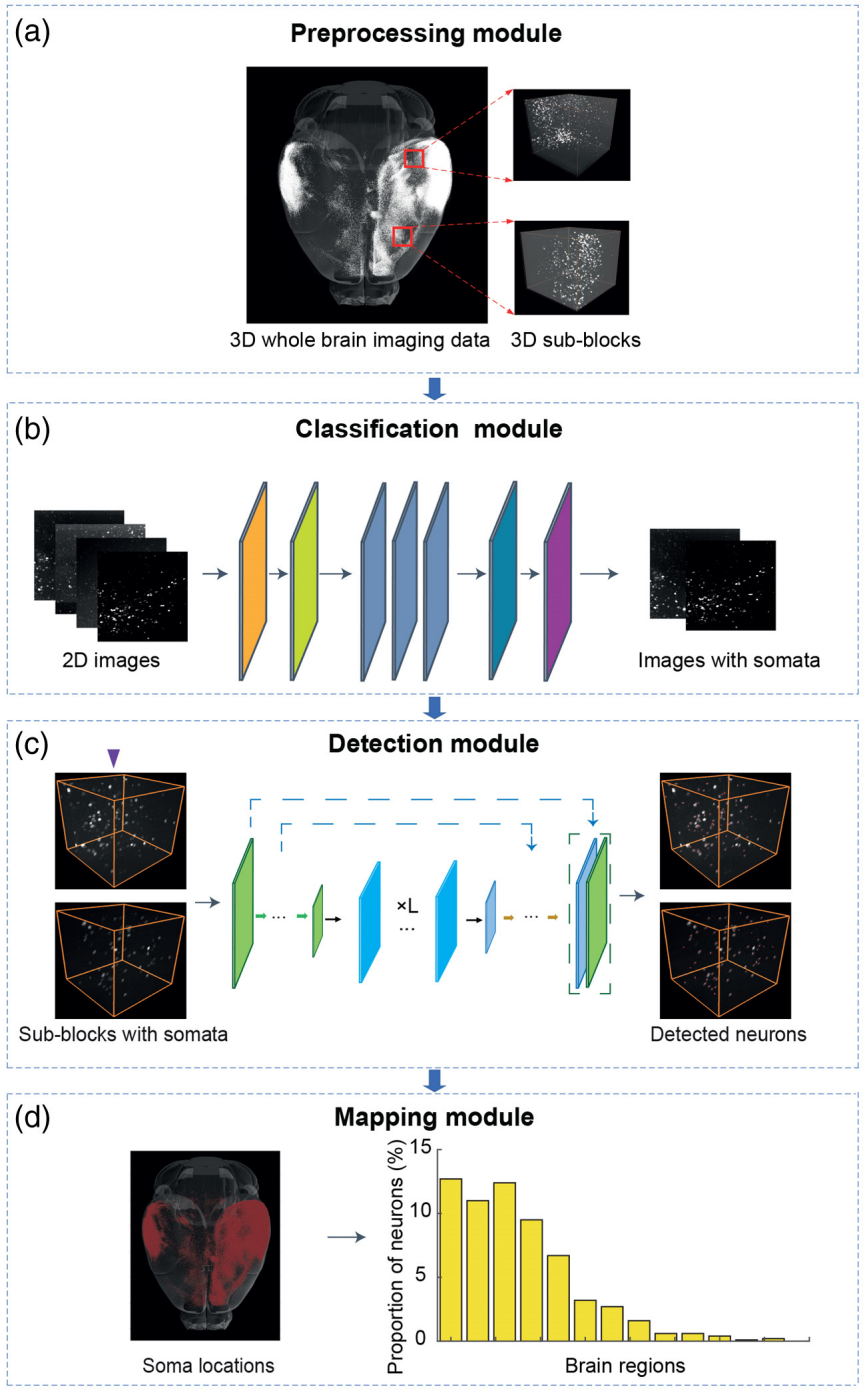
Processing pipeline of the proposed 3D whole-brain neuron detection and mapping. (a) A 3D whole-brain imaging data is first processed into multiple sub-blocks and then further converted into 2D MIP images which serve as the input for classification. (b) The classification module identifies the images that contain soma information and further tags the corresponding 3D sub-blocks. (c) The detection module follows the tag and segments the soma regions in the corresponding sub-blocks, and the detected coordinates are obtained by taking the center points of the soma regions. (d) The coordinates of the detected neurons are transformed into the Allen Brain Atlas coordinate system (left). The detected neurons are counted automatically and the number of neurons in each brain region is divided by the total in the whole brain (right).

Our approach is devised to enable high-throughput detection and analysis of neuronal somata, conforming to a structured workflow (Fig. 2). This process commences with the preparatory stage of transforming 3D whole-brain imaging data into smaller, manageable sub-blocks, and then generating 2D MIP images at this stage [Fig. 2(a)]. The subsequent phase involves the classification module [Fig. 2(b)], where MIP 2D images are assessed to identify those carrying soma information. Once classified, the detection module employs a Video Swin Transformer–based segmentation network to process the corresponding 3D sub-blocks. This network segments the soma regions within these sub-blocks [Fig. 2(c)]. After segmentation, a connected domain analysis is conducted to determine the precise coordinates of each detected neuron. Finally, the mapping module integrates the soma spatial information, correlating neuron positions with a 3D brain atlas. This mapping assigns each neuron to a specific brain region [Fig. 2(d)]. In total, the proposed approach represents an integrated technique for soma detection and analysis in large-scale 3D brain imaging datasets, enabling efficient investigations of neuronal structure patterns.

During the training and inference stages of the NeuronMapper framework, the time performance of each component is summarized as follows: For the classification task, training proceeds at a rate of 4.5 s per epoch with a batch size of 8, whereas inference operates at a speed of 10.2 ms per sample using the same batch size. Regarding segmentation, training occurs at a pace of 12 s per epoch when using a batch size of 1, and inference is performed at a rate of 91.7 ms per sample with the batch size maintained at 1.

### 3.3 Identification of Images Containing Somata Within Whole-Brain Imaging Data

Considering the abundant and complex soma information generated by whole-brain imaging, the precision by which a method can identify somata turns into a crucial factor. In our quest to enhance computational efficiency, we initiated by converting the 3D sub-blocks into 2D MIP images, a step that is crucial for the classification process. We designed a classification network based on the principle of ResNet architecture, including an initial convolutional layer that is responsible for increasing the image channel dimensions.^34^ After this, the network expands through four residual blocks, along with a pooling layer, which jointly works to extract the core features of the images while prudently reducing the number of parameters, ensuring that no essential image characteristics are compromised in the process (Fig. S1 in the Supplementary Material). The detailed design of each layer within the classification network is described in Table S2 in the Supplementary Material.

**Fig. 3:**
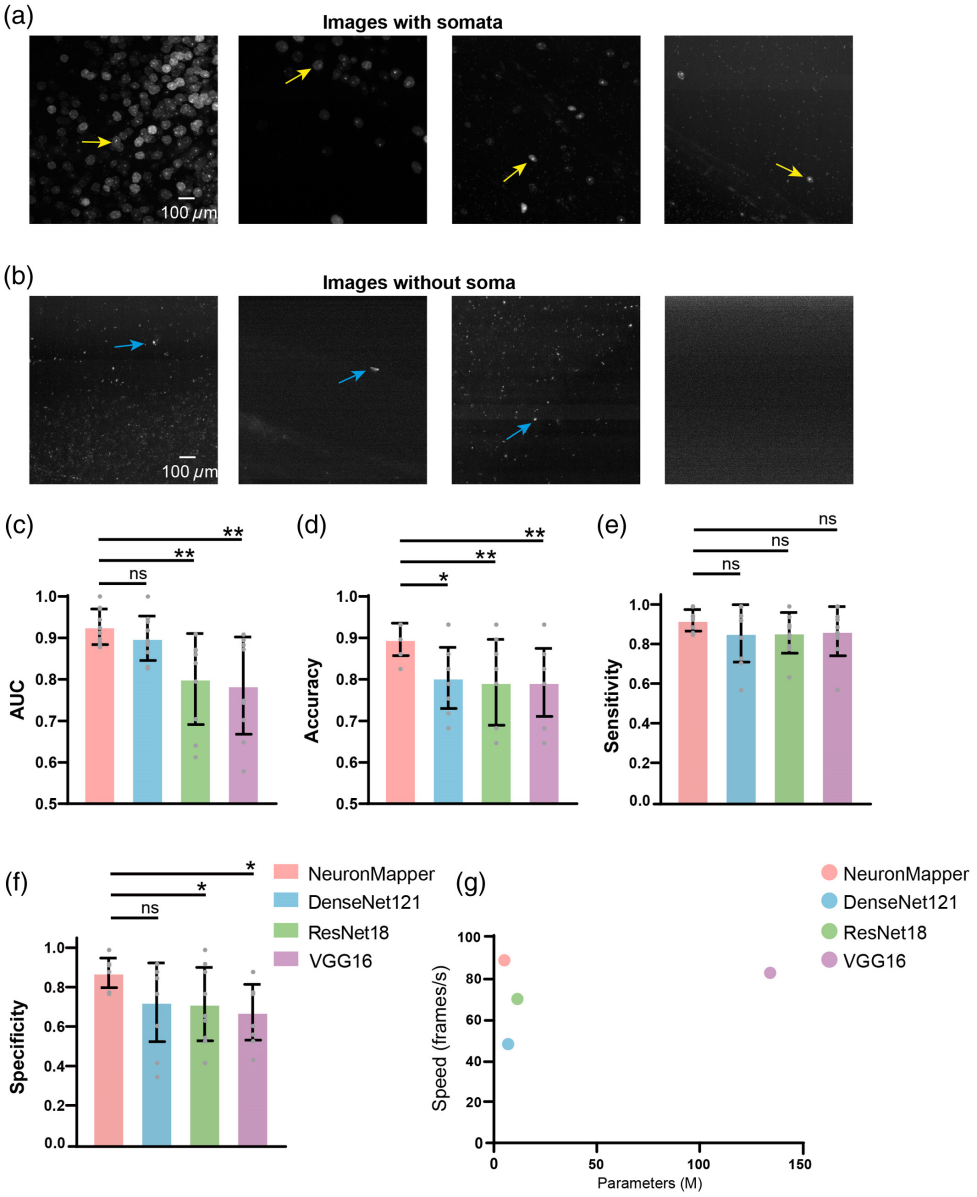
Representative results of classification and comparison with other methods. (a) 2D MIP images are classified as containing soma information. (b) 2D MIP images are classified as not having soma information. Yellow arrows indicate somata in the images and blue arrows imply background noises. (c)–(f) Comparison of our classification module with other methods in terms of the AUC (c), accuracy (d), sensitivity (e), and specificity (f). The score for each random test is represented as a gray dot. *p<0.05; **p<0.01; n=10 images; two-sided Wilcoxon signed-rank test; error bars are SD. (g) Comparison of our classification module with other methods in terms of processing speed and parameters.

To validate the performance of our classification module, we assembled a dataset that consists of 180 exemplary sub-blocks, which were carefully selected to reflect a wide range of soma densities, diverse fluorescence intensities, and instances of background noise. This dataset was randomly divided as: 120 sub-blocks were designated for training, 30 sub-blocks for validation, and 30 sub-blocks for testing. This random assignment was repeated 10 times, and the performance metrics were averaged over the 10 outcomes. Figure 3(a) illustrates four representative classification results, featuring different soma distribution patterns, along with images that solely consist of background noises [Fig. 3(b)]. Furthermore, we conducted a performance comparison involving our classification module and other three deep neural networks, namely, DenseNet121, ResNet18, and VGG16.^34^^–^^36^ All compared neural networks were trained on the same dataset as NeuronMapper, ensuring that the weights were learned within our study. The preprocessing steps and other methodologies were directly applied from previous studies. We first assessed the models’ capabilities using two performance indicators—the AUC [Fig. 3(c)] and accuracy scores [Fig. 3(d)]. Our classification module yielded promising results, achieving an AUC of 0.93 and an accuracy of 0.90. In comparison, when evaluated on the same whole-brain imaging dataset, no other neural networks attained an AUC exceeding 0.90 or an accuracy surpassing 0.80 [Figs. 3(c) and 3(d)]. Moreover, to ensure that the recognition methods are both sensitive and specific, we evaluated sensitivity [Fig. 3(e)] and specificity [Fig. 3(f)]. Our proposed network exhibits superior sensitivity and specificity compared with other methods, although some of these differences do not reach statistical significance. In addition, our classification module achieved the smallest size of model parameters and the fastest speed among the tested methods [Fig. 3(g)]. This significant disparity clearly demonstrates the superior performance of our classification module in accurately identifying sub-blocks with somata in the intricate pattern of whole-brain imaging data.

### 3.4 Segmentation of Somata Within 3D Imaging Data

Upon the completion of the classification stage, the image blocks that were verified to contain somata underwent indexing, which enabled the detection module to selectively target the relevant 3D sub-blocks in accordance with the established index. The design of the segmentation network aimed to maximize the utilization of both high-level semantic information and detailed low-level features, which was accomplished through the strategic combination of convolutional layers with the U-Net architecture. This architecture is characterized by its sequence of encoder and decoder layers, facilitating the preservation of spatial context during the segmentation process. To further enhance the segmentation’s accuracy, especially in dealing with long-range dependencies and incorporating comprehensive contextual information, the module incorporated the Video Swin Transformer. This integration of the structural advantages of U-Net with the transformer’s prowess in handling complex spatial relationships was specifically intended to reduce occurrences of incomplete or inaccurate segmentations, thereby enabling significantly more refined and precise somata boundaries [Fig. S2(a) in the Supplementary Material]. Figure S4 in the Supplementary Material depicts representative examples of the segmentation outcomes generated by the segmentation network and other four deep learning segmentation networks. These comparisons clearly reveal the enhanced precision and robustness of our segmentation network, particularly in addressing challenges such as nonuniform brightness and incomplete somatic details. In the task of segmenting somata, our network consistently outperformed SegNet, 3D U-Net, ResUNet, and TransBTS, providing more reliable segmentation results.^37^^–^^40^ The competing methods frequently overlook soma information to varying degrees, potentially degrading the accuracy of subsequent coordinate acquisitions [Figs. S4(a) and S4(b) in the Supplementary Material]. Notably, when dealing with sub-blocks that contain a single soma among elements, our segmentation module demonstrated remarkable reliability. It effectively segmented the soma while simultaneously excluding similar noises. By contrast, the other methods incorrectly segmented the noises to different extents [Fig. S4(c) in the Supplementary Material].

For a more comprehensive understanding, Fig. S5 in the Supplementary Material provides a 2D view through the 3D segmentation results across a range of conditions. In sub-blocks marked by unevenly distributed fluorescence intensity, our segmentation module had better performance than the other methods. For example, 3D U-Net, ResUNet, and TransBTS only partially segmented the soma [Fig. S5(a) in the Supplementary Material]. In situations where sub-blocks had incomplete soma morphology, our segmentation network was able to accurately delineate the soma regions. Meanwhile, SegNet exhibited deficiency by missing the soma [Fig. S5(b) in the Supplementary Material]. In the challenging situation of sub-blocks with weak fluorescence signals, our approach achieved the highest level of segmentation accuracy among all the evaluated methods [Figs. S5(c)–S5(e) in the Supplementary Material]. By contrast, the competing methods often failed to locate neurons in various circumstances.

### 3.5 Comparison of Neuron Segmentation and Detection Methods

**Fig. 4:**
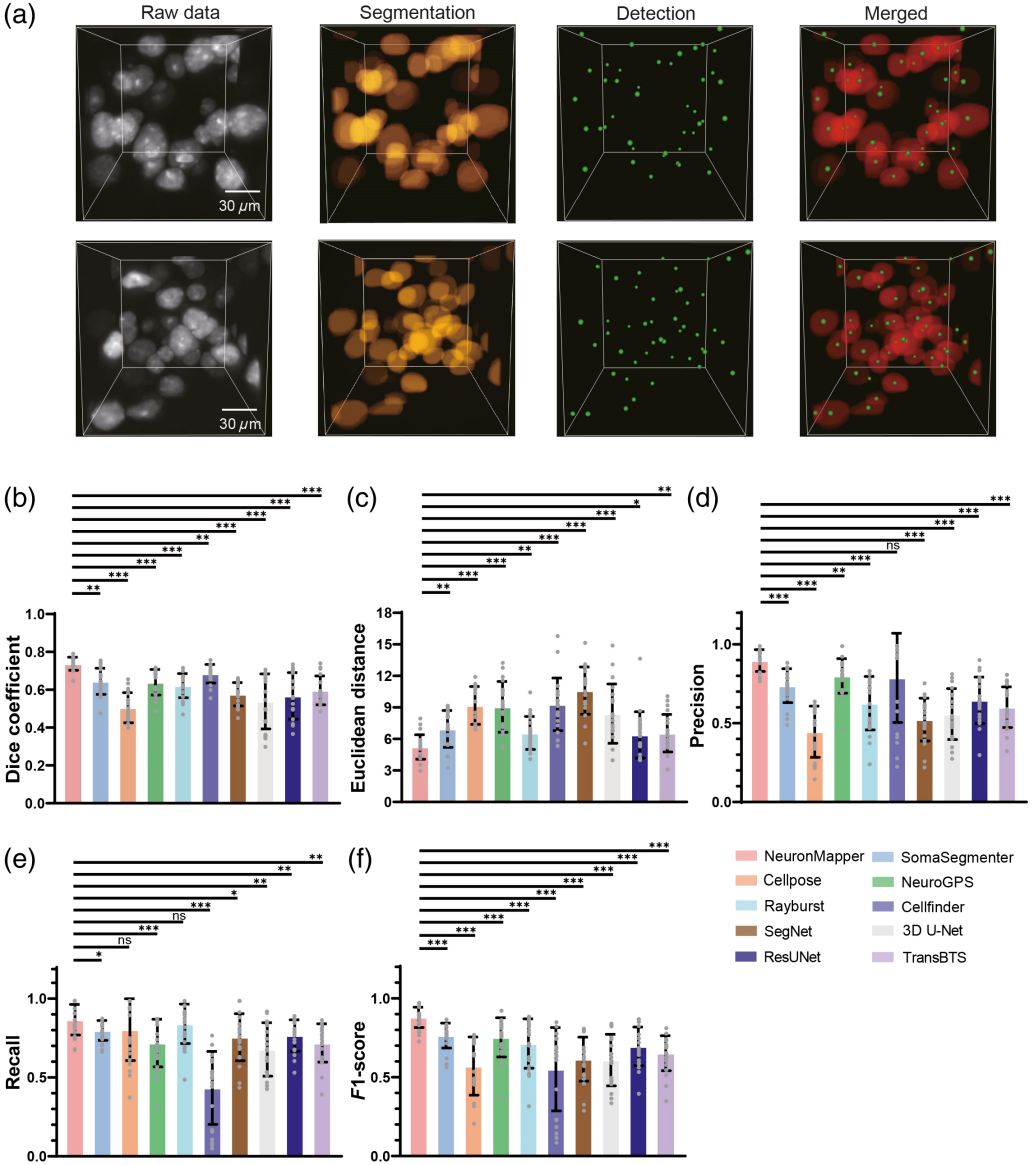
Comparison of soma segmentation and detection methods. (a) Two representative detection processes from raw data to segmented and detected results are presented at the sub-block level. (b)–(f) The comparison between our approach and other methods (SomaSegmenter, Cellpose, NeuroGPS, Rayburst, Cellfinder, SegNet, 3D U-Net, ResUNet, and TransBTS) for segmentation metrics, including Dice coefficient (b), Euclidean distance (unit: pixels) (c), and detection metrics, including precision (d), recall rate (e), and F1-score (f). The score for testing each image block is indicated as a gray dot. *p<0.05; **p<0.01; ***p<0.001; *ns*, not significant; n=20 image blocks; two-sided Wilcoxon signed-rank test; error bars are SD.

Following the image segmentation, a subsequent connected domain analysis was carried out to locate and extract the precise centroid coordinates of each segmented neuronal soma. Figure 4(a) shows two representative examples that highlight the raw data, the segmentation of soma structures, and the detection of their exact locations at the sub-block scale. These demonstrations clearly verify the robustness of our approach in accurately recognizing and spatially mapping neurons within the 3D image data while simultaneously ensuring an outstanding level of consistency and reliability when applied to various sub-block regions.

To further validate the capability of our approach, we carried out comparisons against nine other methods: SomaSegmenter (available at https://github.com/lens-biophotonics/SomaSegmenter), Cellpose, NeuroGPS, Rayburst, Cellfinder, SegNet, 3D U-Net, ResUNet, and TransBTS, for neuron segmentation and detection.^9^^,^^13^^,^^14^^,^^18^^,^^37^^–^^40^ The statistical comparison results are summarized in Figs. 4(b)–4(f). In the tests, we used image sub-blocks containing different distribution patterns of somata and performed the segmentation and detection processing. Our initial emphasis was also on the segmentation fidelity, and thus, we quantified the Dice similarity coefficient and Euclidean distance for our proposed method and the competing approaches. The results revealed a significant disparity in favor of our approach and a significantly and statistically higher Dice coefficient [Fig. 4(b)], which was in sharp contrast to those of Cellpose. Furthermore, the Euclidean distance measure of our approach shows the lowest values [Fig. 4(c)], also suggesting better segmentation performance than the other methods. These results demonstrated the superiority of our method in the segmentation of neuronal somata. After that, we performed an assessment of the efficacy of neuron detection. Using the metrics of precision, recall rate, and F1-score, we evaluated the detection performance of each method. The comprehensive evaluation confirmed that our approach clearly outperformed the competing methods, boasting a significantly higher precision, recall rate, and F1-score [Figs. 4(d)–4(f)]. This remarkable performance attests to the reliability and effectiveness of our method.

Given the challenges posed by overlapping cells for segmentation, we examined such cases and compared the segmentation performance (Fig. S6 in the Supplementary Material). A representative example clearly demonstrates that NeuronMapper provided superior segmentation quality, whereas other methods did not match our results. Specifically, Cellpose and Rayburst missed many somata during segmentation, and the other methods either failed to capture certain regions of somata or produced lower-quality segmentations.

In addition, as NeuronMapper is a multistage approach that includes an initial classification stage to exclude images not requiring further analysis, we conducted a test to validate the role of this classification module. We compared the performance of our method both with and without the classification module to assess its impact. The detection results, shown in Fig. S7 in the Supplementary Material, indicate that there is no significant difference in performance whether the classification module is used or not. Specifically, for images lacking somata, our detection module seemed to not generate noticeable false positives. Therefore, these findings suggest that the inclusion of the classification stage did not appreciably affect the overall accuracy.

### 3.6 Generalization Testing of Classification and Detection Methods

**Fig. 5:**
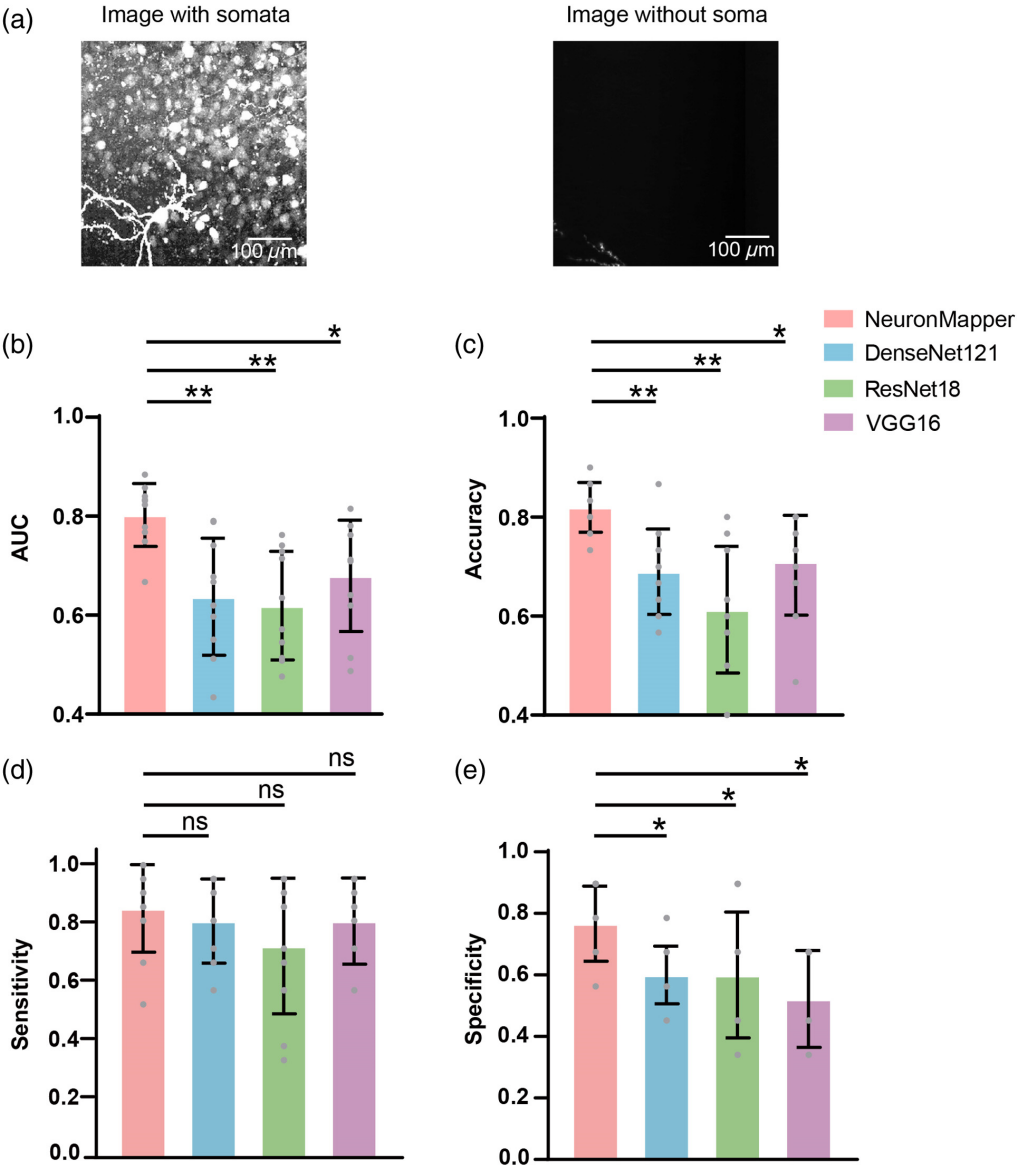
Comparison with other methods for classification in public dataset. (a) 2D MIP images are classified as containing soma information and not having soma information. (b)–(e) Comparison of our classification module with other methods in terms of the AUC (b), accuracy (c), sensitivity (d), and specificity (e). The score for each random test is represented as a gray dot. *p<0.05; **p<0.01; ***p<0.001; ns, not significant; n=10 images; two-sided Wilcoxon signed-rank test; error bars are SD.

To further validate the generality and stability of our method, we employed publicly available brain imaging datasets from mice for testing our classification, segmentation, and detection algorithms. The classification images were sourced from the Allen Brain Institute’s fMOST imaging system, specifically using three mouse brain images with IDs 321237-17302, 321244-17545, and 373368-18455.^22^^,^^41^ For this study, we selected 30 3D sub-blocks from these datasets and converted them into 2D images using slicing and MIP, creating a dataset that included both images with and without somata for generalization testing. We then applied the classification networks, previously trained on our lab’s datasets, to infer the presence or absence of somata in these unseen brain images [Fig. 5(a)]. Although there was a slight performance drop compared with the results shown in Fig. 3, this is attributed to the classification networks not having been retrained on the new public dataset. Despite this, as illustrated in Figs. 5(b)–5(e), NeuronMapper consistently outperformed the other three methods across various performance metrics, aligning with our earlier findings.

**Fig. 6:**
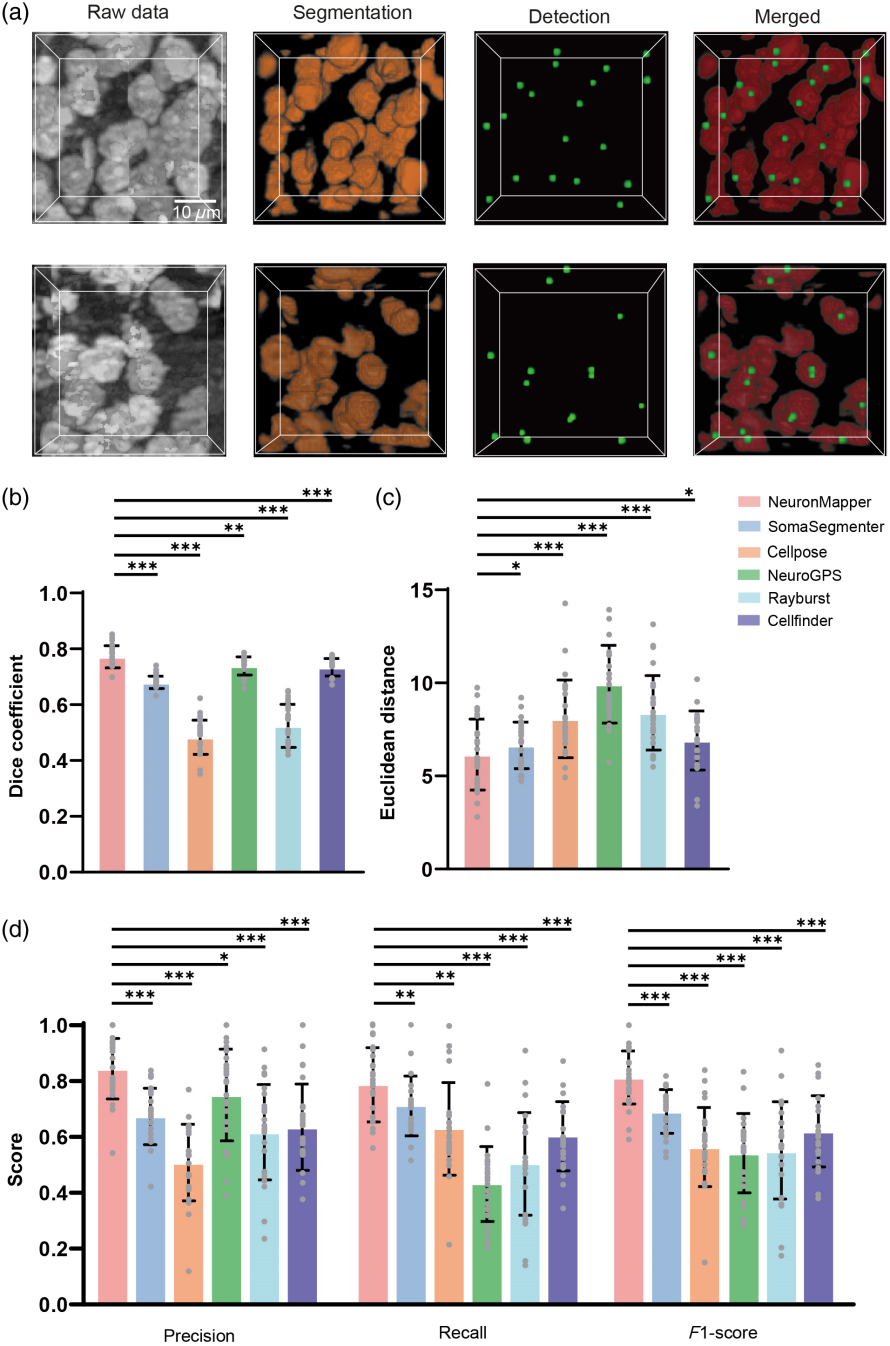
Comparison with other methods for segmentation and detection in public dataset. (a) Two representative detection processes from raw data to segmented and detected results are presented at the sub-block level. (b)–(d) The comparison between our approach and five other methods for segmentation metrics, including Dice coefficient (b), Euclidean distance (unit: pixels) (c), and detection metrics, including precision, recall rate, and F1-score (d). The score for testing each image block is indicated as a gray dot. *p<0.05; **p<0.01; ***p<0.001; ns, not significant; n=25 image blocks; two-sided Wilcoxon signed-rank test; error bars are SD.

In addition, we applied our detection module to a Nissl-stained brain imaging dataset acquired using the micro-optical sectioning tomography system,^21^ specifically utilizing data from ID 01 (https://github.com/keepersecond/neuronal-soma-segmentation/releases/). This dataset was divided into 25 sub-blocks, each with dimensions of 128×128×86  voxels (0.35×0.35×0.35  μm/voxel), to form a segmentation dataset for generalization testing. Given that the original dataset lacked ground truth (GT) annotations, we relabeled it. Two experienced annotators independently labeled each neuron and subsequently compared their results to reach a final consensus for the GT. After training the models on our dataset, we applied them to the unseen Nissl-stained data. The representative examples in Fig. 6(a) demonstrate that our approach achieved good performance in both segmentation and detection. By comparing performance metrics for segmentation (Dice coefficient and Euclidean distance) and detection (precision, recall, and F1-score), it is evident that NeuronMapper outperformed SomaSegmenter, Cellpose, NeuroGPS, Rayburst, and Cellfinder [Figs. 6(b)–6(d)]. Consequently, NeuronMapper consistently delivers reliable and robust soma segmentation and detection results.

### 3.7 Brain-Wide Neuron Detection and Mapping

**Fig. 7:**
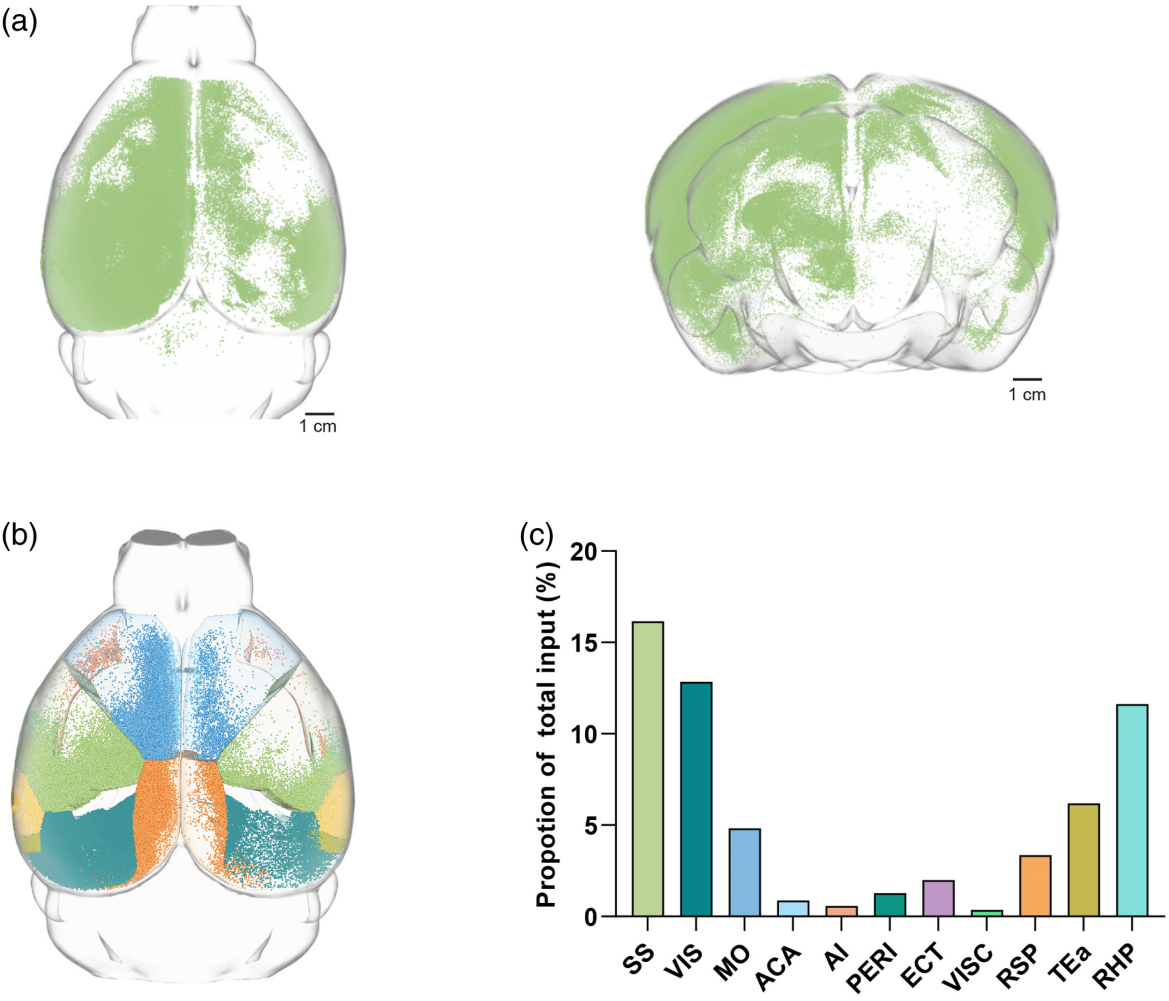
Brain-wide soma detection and mapping based on the proposed method. (a) The 3D whole-brain neuron detection result (1,231,906 detected neurons, mouse #1) is shown in horizontal (left) and coronal (right) views. (b) A 3D view of the detected somata in the whole-brain atlas, with the brain regions being marked by different colors. (c) Quantitative analysis of input neurons in different brain regions of the mouse is shown in panel (a). SS; somatosensory area; VIS, visual areas, MO, somatomotor areas; ACA, anterior cingulate area; AI, agranular insular area; PERI, Peririnal area; ECT, Ectorhinal area; VISC, visceral area; RSP, retrosplenial area; TEA, temporal association areas; RHP, retrohippocampal region.

We then performed whole-brain soma detection, with the detected individual soma over a million level (e.g., 1,231,906 detected neurons from mouse #1, Table S1 in the Supplementary Material). A 3D visualization of the total input neurons within a mouse brain is exhibited in Fig. 7(a).

To demonstrate the capabilities of our approach developed for 3D whole-brain neuron mapping, we applied our method to the whole-brain imaging datasets for the detection and mapping of somata (Table S1 in the Supplementary Material). With the detected somata, we proceeded to map neurons with registration and perform quantitative analyses for the different brain regions [Fig. 7(b)]. The integration of whole-brain atlas and quantitative analysis revealed the distribution of neurons in a series of brain regions while simultaneously uncovering the interesting ipsi-contra bias shown by input neurons [Fig. 7(c)]. This comprehensive analysis thus provides a profound insight into the underlying neuroanatomical architecture, highlighting the potential of our approach in advancing neuroscience research.

## 4 Discussion

Mapping of the spatial distribution of specific neurons is essential for understanding the neural circuits that are related to various brain functions.^42^^,^^43^ Developing accurate and fast soma detection techniques is able to accelerate our quest for understanding the brain.^44^^,^^45^ However, the innovations of whole-brain imaging techniques have brought about high-resolution, large-scale data that far exceeds the capacity of manual labeling.^46^ To address this dilemma, we present an automated approach designed to analyze labeled somata within whole-brain imaging data. The proposed approach initiates with dividing whole-brain data into smaller, more manageable sub-blocks, and transforming them into MIP 2D images. Then, a classification network is used to screen these images to accurately identify those containing somata and tag the associated sub-blocks. Subsequently, a segmentation network uses the tags to precisely segment the soma regions within the corresponding sub-blocks. Based on these segmented areas, the centroid coordinates are extracted for whole-brain and registered with Allen Brain Atlas for neuron mapping analyses.

Our approach provides a solution to the following issues: first, our classification module is devised to distinguish the MIP 2D images that contain somata from those that do not. Our classification network is designed as a four-stage network structure. This network structure is shallow, and each layer has a single basic block, which makes the network model simple and lightweight (Fig. S1 in the Supplementary Material). In comparison to deeper networks such as ResNet18 and DenseNet121, our network parameters and computational complexity are smaller, reducing the risk of overfitting during classification training. Each basic block contains two convolution layers and a shortcut connection that directly adds the input to the output without changing the feature dimension. This connection helps gradient propagation, avoids the issue of gradient disappearing, makes the feature extraction process efficient, and can well retain the original information while extracting effective high-level features. Regarding down-sampling, we utilized a convolution layer with a step length of 2. Compared with VGG16, which employs the maximum pooling layer for down-sampling, the convolution layer can retain more information for down-sampling, which is conducive to enhancing the performance of classification tasks. The results strongly confirm the superiority and effectiveness of our approach in accurately identifying sub-block images with the presence of soma (Fig. 3).

Second, in contrast to previous studies that mainly focus on brain slices,^19^^,^^47^^,^^48^ our approach directly processes soma information within 3D whole-brain image data (Fig. 4). Our segmentation network exhibits exceptional precision in outlining soma regions, with few cases of misclassification or segmentation incompleteness. In brain-wide 3D neuron detection and mapping, a single neuron can span multiple tiles, with the information contained in a single tile often being insufficient to capture the neuron’s boundary continuity or global morphology. This insufficiency can lead to a single neuron instance being fragmented into multiple parts during segmentation. Our method addresses this challenge by leveraging SW-MSA and a hierarchical structure to build both local and long-range dependencies. By repeatedly applying self-attention modules within this framework, the network establishes cross-tile dependencies at deeper layers and across larger spatial extents. Following this, we employ connected-component analysis to consolidate instance segmentation results at tile boundaries, ensuring each neuron receives a unified label. This approach effectively prevents neuronal fragmentation, thereby enhancing the accuracy and integrity of the segmentation.

We tested our NeuronMapper against other neural methods, including SomaSegmenter, Cellpose, NeuroGPS, Rayburst, Cellfinder, SegNet, 3D UNet, ResUNet, and TransBTS. Comparative evaluations of segmentation outcomes are shown in Figs. 4 and 6 and Figs. S4–S6 in the Supplementary Material, demonstrating our network’s accuracy even under adverse imaging conditions such as fluctuating brightness levels, the presence of a contaminant, and discontinuous soma data. The strategic integration of the Video Swin Transformer within our segmentation network substantially enhances the handling of long-range dependent information, a capability that is often lacking in traditional CNN models.^49^ Despite the traditional dominance of CNNs in biological image segmentation, their inherent limitations in capturing the global context have hindered their full potential. On the contrary, the Swin Transformer, distinguished by its computation through shifted window mechanisms, has proven its effectiveness in various applications, including noise reduction^50^ and tumor segmentation,^51^^,^^52^ and most recently, a study highlighted its successful use in two-photon imaging data processing.^53^ However, these applications were confined to 2D segmentation paradigms. Notably, a recent contribution emphasized the utilization of Swin Transformers for 3D segmentation tasks in brain tumor MRI datasets, although not at the cellular resolution.^54^ Complementing this, the inclusion of skip connections facilitates the retention of high-resolution image details throughout the segmentation process, ensuring that no detail is overlooked in the pursuit of precision.^55^ Based on these improvements, our segmentation network provided unbiased and reliable segmentation similar to manual annotations for 3D image sub-blocks.

Given the ongoing challenges in processing complex brain imaging data across various scenarios,^33^ we demonstrated the generalizability of our trained models by evaluating their classification, segmentation, and detection capabilities on new, unseen public mouse brain imaging datasets. Although dataset division somewhat affected performance, the comparison results (Figs. 5 and 6) show that NeuronMapper maintains robust generalization capabilities. Our model exhibits consistently high performance in classification, segmentation, and detection tasks, even when applied to imaging data from different laboratories. Moving forward, we aim to further enhance our approach to improve model generalizability and performance across diverse datasets.

Finally, our approach uses connected domain analysis to identify and locate the somata in three-dimensional space. By analyzing the segmented results, the geometric center of each soma was determined and regarded as its coordinate. These coordinates were then integrated to enable the visualization and exploration of the distribution of somata at both the sub-block and whole-brain levels. The 3D visualization of somata provides a valuable understanding of the overall organization of the neurons in the brain. Advancements in neuroscience technologies have positioned transsynaptic viral tracers as a powerful tool for mapping functional neural circuits. Retrograde transsynaptic viral tracers, such as rabies virus and pseudorabies virus, have been widely utilized to delineate the presynaptic inputs to neurons that have been transduced. Therefore, we introduced nuclear-locating elements into our labeling strategy and combined retrograde and monosynaptic anterograde viral labeling methods with high-resolution whole-brain imaging to uncover the brain-wide fine connectivity structures. By categorizing neurons based on their spatial location and distribution, it allows screening of functional neural connectivity embedded in complex brain circuits. This enabled us to examine the quantitative, target-specific, and layer-specific characteristics of the neural input and output connectivity. In Fig. 7(a), asymmetry in neuronal density can be observed between the two hemispheres. The results indicate that for both input and output connections, nearly all regions were ipsilateral-biased to the side of virus injection, with neuronal density predominantly observed in the ipsilateral hemisphere, which is consistent with the previous studies.^3^^,^^56^ Researchers can observe the spatial arrangements of somata, identify spatial patterns or clustering, and acquire a deep understanding of the brain’s intricate connectivity. By mapping the detected soma to specific brain regions [Figs. 7(b) and 7(c)], it has the potential to uncover new aspects of insight into the functional circuitry and the underlying topology of neural networks, promoting the field of neuroscience toward a deeper understanding of brain functions.^57^^,^^58^

Although NeuronMapper has demonstrated success in precisely segmenting the regions of neuronal somata at the whole-brain level, it encounters challenges when it comes to accurately segmenting closely adjacent somata without the usage of post-processing. Even though this limitation did not have a substantial influence on the quantification results presented in the current study, it is an aspect that requires further improvement to overcome the challenging spatial proximity and provide a more effective segmentation of neuronal bodies. Furthermore, we plan to improve the functionalities of the whole-brain registration and brain region analysis. The aim is to provide researchers with a more comprehensive and reliable tool for analyzing input and output neurons in various brain regions. Therefore, we will focus on improving our approach that can seamlessly integrate specific brain region information into the segmentation and quantification processes. This would streamline the analysis workflow, saving researchers’ time and effort while ensuring accurate results.

## 5 Conclusion

In summary, our approach demonstrated its effectiveness in the detection and mapping of soma in whole-brain imaging data. It accurately distinguished soma regions from background noises, performed precise segmentation, and generated reliable soma locations for mapping to diverse brain regions. This brain-wide neuron detection and mapping capability enhances our understanding of brain connectivity, neural circuitry, and underlying computational processes.

## Supplementary Material

10.1117/1.NPh.12.2.025012.s01

## Data Availability

The code of NeuronMapper is freely available at https://github.com/XZH-James/NeuronMapper. Data underlying the results presented in this paper are not publicly available but may be obtained from the corresponding author Xiang Liao (xiang.liao@cqu.edu.cn) upon reasonable request.
