# Supplementary material for: Brain-wide 3D neuron detection and mapping with deep learning
Source: Neurophotonics. 2025 May 20;12(2):025012. doi: 10.1117/1.NPh.12.2.025012 (PMC12093273; doi:10.1117/1.NPh.12.2.025012)
Supplement: Supplementary file 1 [file NPh_012_025012_SD001.pdf]

Supplemental Material

Brain-wide 3D neuron detection and mapping with deep learning

Yuanyang Liu, Ziyan Gao, Zhehao Xu, Chaoyue Yang, Pei Sun, Longhui Li, Hongbo Jia, Xiaowei Chen, Xiang Liao, Junxia Pan, and Meng Wang

Supplemental Figures

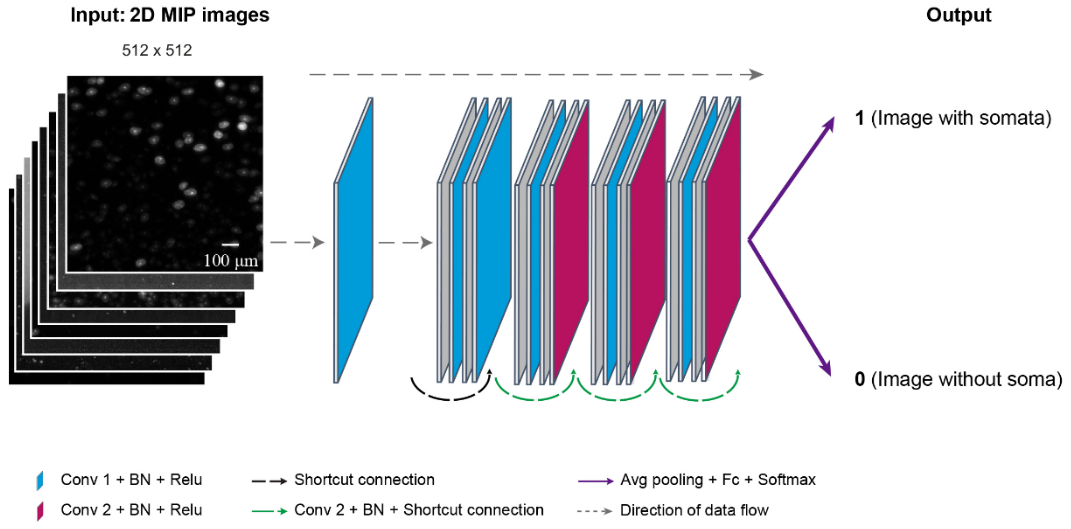

Fig. S1 Classification network used for identifying the images with soma. The blocks and arrows represent different processing layers. The blue and violet blocks denote convolutional layers with different strides, represented as conv1 and conv2, respectively. Both the kernel sizes of conv1 and conv2 are 3 × 3. The down-sampling layer is conv2 with a stride of 2. The purple arrow represents the stacking of average pooling, Fc and Softmax function. The gray dotted arrow indicates the direction of data flow, while the green and black dotted arrows represent shortcut connections with or without the 1 × 1 convolution layer, respectively. Abbreviation: Conv: Convolutional layer; BN: Batch Normalization; Avg: Average; Fc: Fully connected layer; Relu: Rectified Linear Unit.

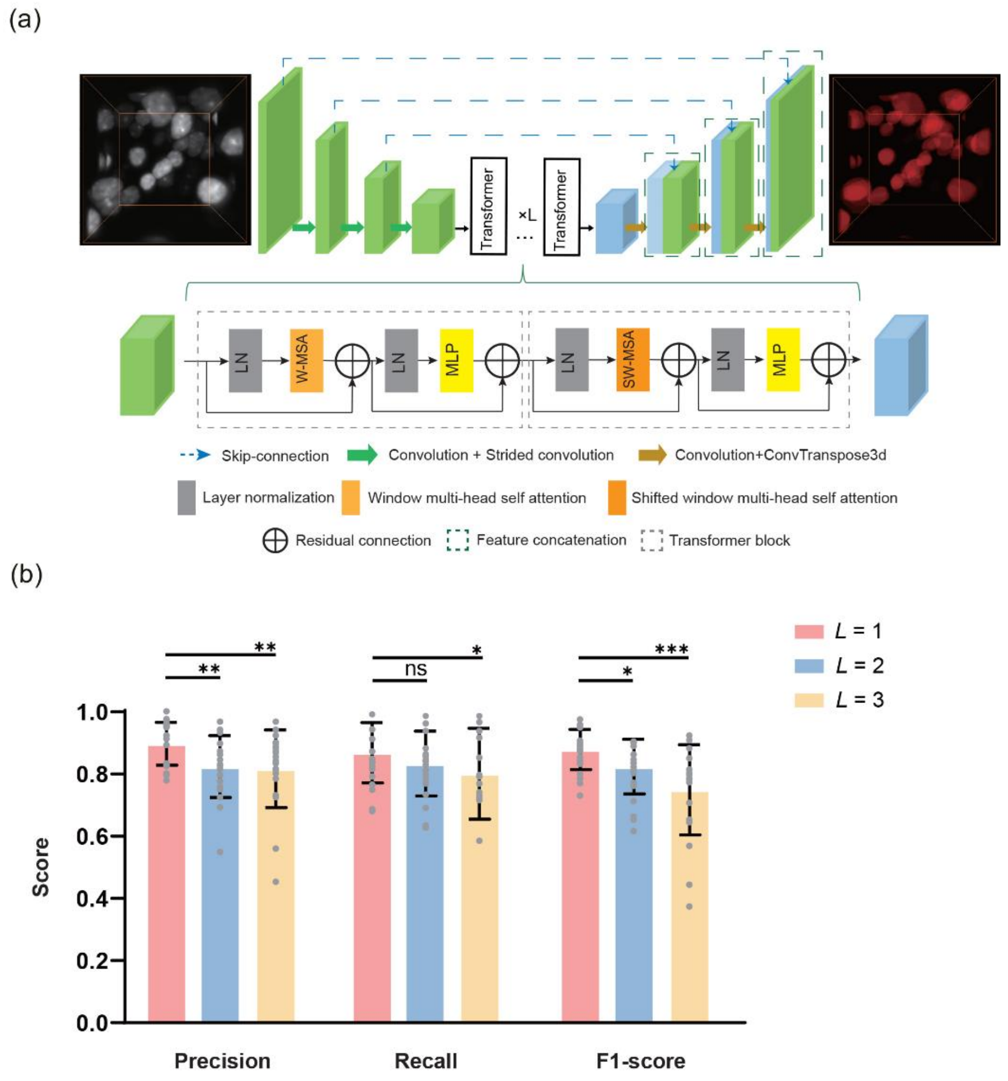

Fig. S2 Segmentation network used for segmenting somata. (a) Illustration of the segmentation network architecture. The colored cubes represent the different process layers within the segmentation network. The green and blue blocks represent convolutional encoder layers and decoder layers, respectively. The green and yellow arrows represent the down-sampling and up-sampling layers, respectively. Both the down-sampling and up-sampling layers are convolutional layers with a stride of 2. The blue arrow indicates the skip connection. The transformer layer consists of L Video Swin Transformers. W-MSA stands for window multi-head self-attention module, and SW-MSA represents shifted window-based multi-head self-attention module. $LN(*)$ is the layer normalization. (b) The comparison evaluates the impact of using different numbers of transformers (L) on detection performance, utilizing three metrics: Precision, Recall, and F1-score. The score for testing each image block is indicated as a gray dot. *$p<0.05$; **$p<0.01$; ***$p<0.001$; *ns*, not significant; $n = 20$ image blocks; two-sided Wilcoxon signed-rank test; error bars are SD.

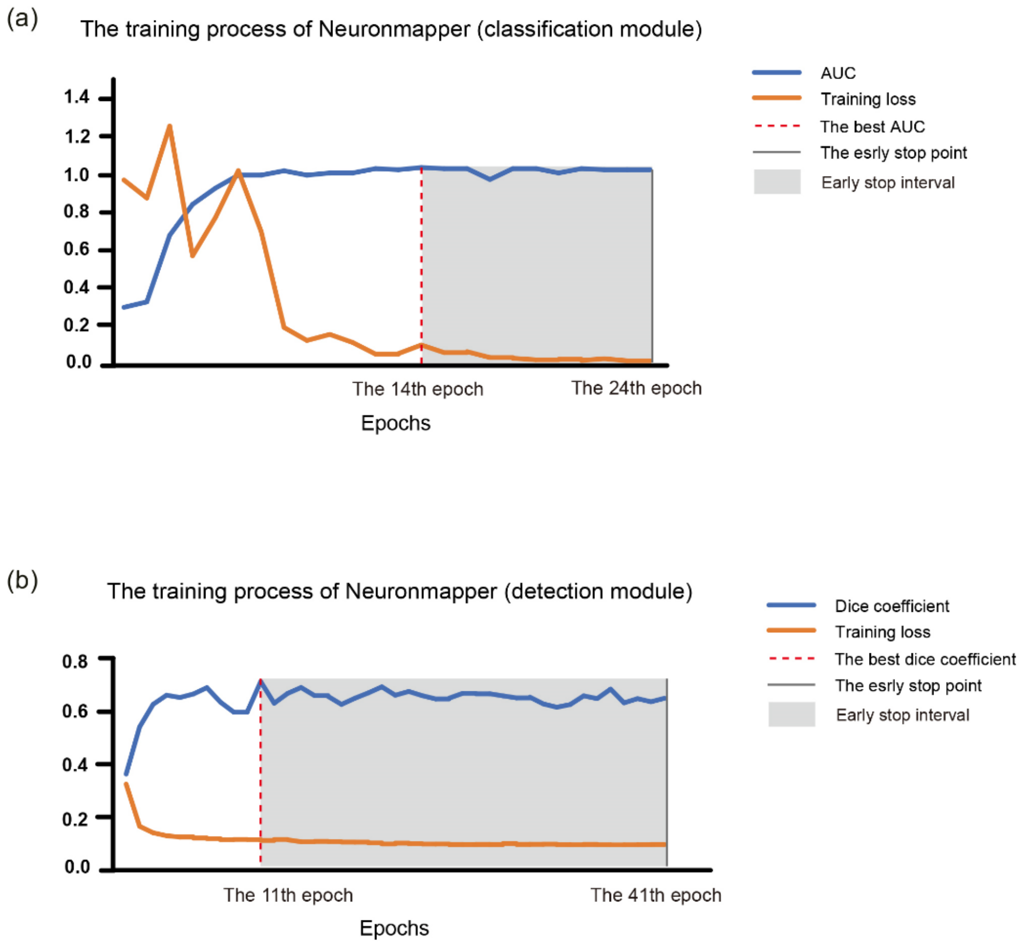

Fig. S3 The training process of NeuronMapper with the early stopping strategy. (a) For the classification module, the blue line represents the AUC on the validation dataset for each epoch during training, while the orange line indicates the training loss. The red dashed line marks the epoch with the highest AUC, and the dark gray line shows the epoch at which early stopping was triggered. The light gray area highlights the 10 consecutive epochs where no significant improvement in AUC was observed. (b) For the detection module, the blue line depicts the Dice coefficient on the validation dataset for each epoch, with the orange line indicating the training loss. The red dashed line marks the epoch with the highest Dice coefficient, and the dark gray line signifies the epoch at which early stopping occurred. The light gray area highlights the 30 consecutive epochs where no significant change in the Dice coefficient was observed.

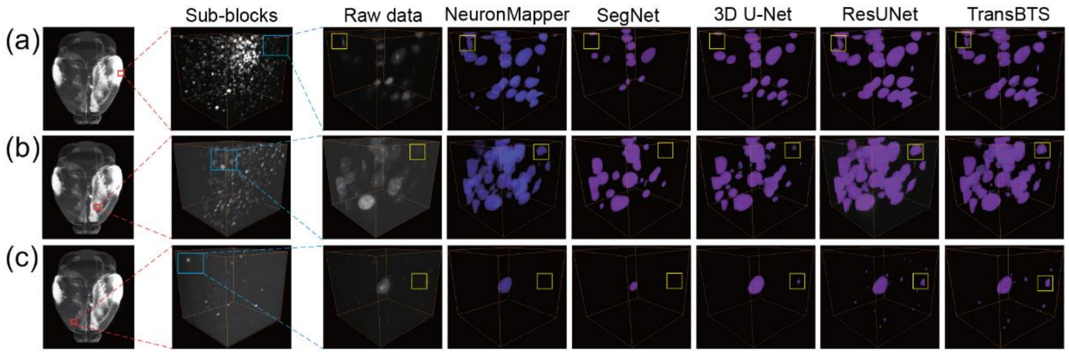

Fig. S4 Comparison between our segmentation network and four other methods. (a) The proposed network segments somata on the edge of a specific sub-block. (b) The segmentation module reliably segments somata with low fluorescence signal. (c) The segmentation network accurately disregards the soma-like noise. The red and blue cubes and dashed lines indicate the location of three illustrating sub-blocks in the dataset. The yellow cubes highlight three different soma conditions.

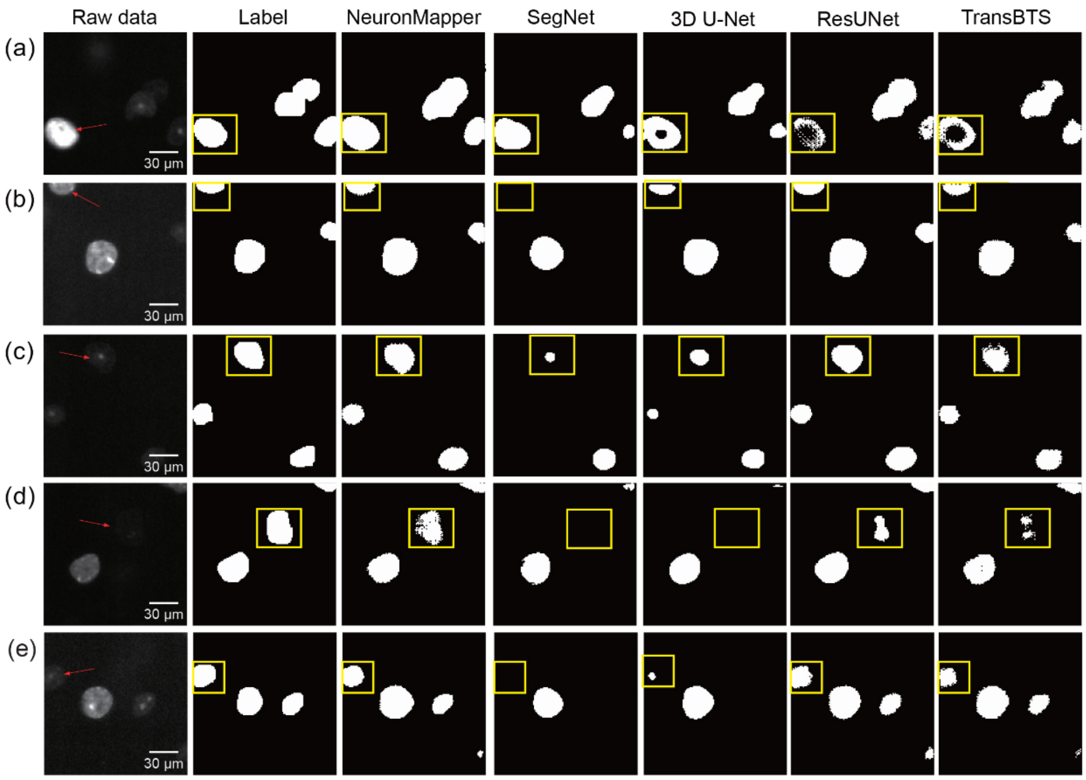

Fig. S5 2D view of the 3D segmentation results for different methods. (a) NeuronMapper provides good segmentation for soma with unevenly distributed brightness. (b) NeuronMapper realizes satisfying segmentation for soma on the edge of the corresponding sub-block. (c-e) NeuronMapper achieves reliable segmentation for somata with low brightness. The red arrows highlight the representative somata. The yellow boxes suggest the corresponding segmentation using different methods.

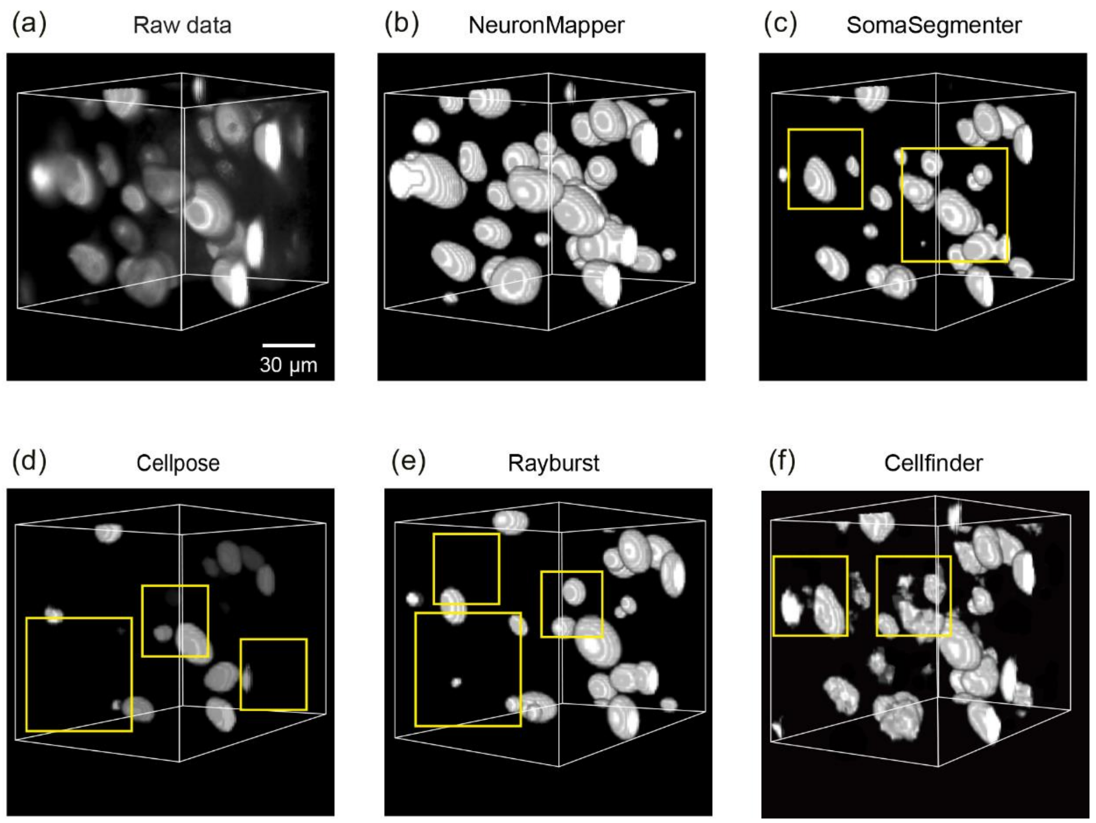

Fig. S6 Representative example of segmentation in the presence of cell overlapping. (a) Raw data of imaged neurons within a sub-block. (b-f) Segmentation results for different methods: NeuronMapper (b), SomaSegmenter (c), Cellpose (d), Rayburst (e) and Cellfinder (f). The yellow box highlights areas where cells are not precisely segmented.

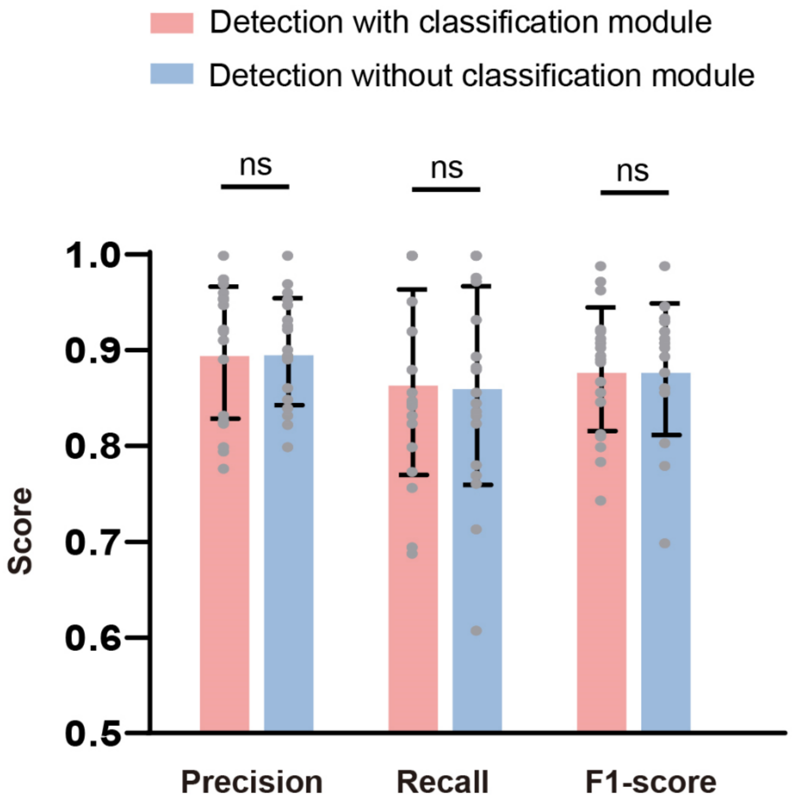

Fig. S7 Comparison of detection results with and without the classification module using three metrics: Precision, Recall, and F1-score. The score for testing each image block is indicated as a gray dot. *ns*, not significant; $n = 20$ image blocks; two-sided Wilcoxon signed-rank test; error bars are SD.

Supplemental Tables

Table S1 Information of whole-brain imaging data used in the experiment

ID	Dimension	Resolution (μm/voxel)	Block number	Number of the detected neurons
mouse #1	28000×20937×3301	0.35×0.35×2	15785	1,231,906
mouse #2	28000×20937×3298	0.35×0.35×2	14590	464,350
mouse #3	30800×22931×5035	0.35×0.35×2	28160	806,898
mouse #4	30800×23000×6198	0.35×0.35×1	35685	1,687,995

Table S2 Details of each layer in the classification network

Layer name	Input size	Layer details	Output size
Layer 1	1, 512, 512	3×3, conv	64, 512, 512
Layer 2	64, 512, 512	3×3, conv+ conv+ shortcut	64, 512, 512
Layer 3	64, 512, 512	3×3, conv+conv2+ shortcut	128, 256, 256
Layer 4	128, 256, 256	3×3, conv+conv3+ shortcut	256, 128, 128
Layer 5	256, 128, 128	3×3, conv+conv4+ shortcut	512, 64, 64
Layer 6	512, 64, 64	Average Pooling	512, 16, 16
Layer 7	131072	Fully Connected	2

Note: Down-sampling is performed by conv2, conv3, and conv4 with a stride of 2.
